# Anthropometric Status among 6–9-Year-Old School Children in Rural Areas in Hai Phong City, Vietnam

**DOI:** 10.3390/nu10101431

**Published:** 2018-10-04

**Authors:** Ngan T.D. Hoang, Liliana Orellana, Tuyen D. Le, Rosalind S. Gibson, Anthony F. Worsley, Andrew J. Sinclair, Ewa A. Szymlek-Gay

**Affiliations:** 1 Institute for Physical Activity and Nutrition (IPAN), School of Exercise and Nutrition Sciences, Deakin University, Melbourne, VIC 3125, Australia; hoangthiducngan@dinhduong.org.vn (N.T.D.H.); anthony.worsley@deakin.edu.au (A.F.W.); 2 Biostatistics Unit, Faculty of Health, Deakin University, Melbourne, VIC 3125, Australia; l.orellana@deakin.edu.au; 3 National Institute of Nutrition, Hanoi 10000, Vietnam; ledanhtuyen@dinhduong.org.vn; 4 Department of Human Nutrition, University of Otago, PO Box 56, Dunedin 9054, New Zealand; rosalind.gibson@otago.ac.nz; 5 Faculty of Health, Deakin University, Geelong, VIC 3216, Australia; andrew.sinclair@deakin.edu.au

**Keywords:** Vietnam, malnutrition, undernutrition, overweight and obesity, abdominal obesity, double burden of diseases, school children

## Abstract

A double burden of malnutrition in Vietnamese children has emerged as a key challenge: childhood undernutrition remains a public health concern while childhood overweight/obesity has gradually increased. This study aimed to (1) estimate the prevalence of undernutrition and overnutrition among 6–9-year-old primary school children in rural areas of Vietnam, and (2) identify sociodemographic factors associated with undernutrition and overnutrition in this population. A cross-sectional survey was conducted in October 2016 in 2334 children from eight primary schools in rural areas in Hai Phong City, Vietnam. Anthropometric and demographic data were collected. The prevalence of underweight, stunting, wasting, and anthropometric failure was 8.0%, 5.1%, 5.3%, and 11.9%, respectively. Up to 22.1% of children were affected by overweight/obesity, and 31.0% by abdominal overweight/obesity. Low maternal education was associated with higher odds of underweight and anthropometric failure, whereas overweight/obesity or abdominal overweight/obesity were more likely in boys and children of mothers with a high education level. This study provides evidence for a double burden of diseases among primary school children in rural areas in Hai Phong City. Future interventions for the prevention and control of childhood undernutrition and overweight/obesity should take into account child sex and maternal education level.

## 1. Introduction

Child malnutrition, which encompasses both undernutrition as well as overnutrition, continues to be a global challenge [1,2]. Recent estimates indicate that up to 25% of preschool children in low- and middle-income countries are at risk of stunting, 15% of underweight, and 8% of wasting [3]. This high prevalence of undernutrition persists into later childhood with up to a third of school children reported as underweight or stunted [4,5,6,7]. Similarly, while overweight and obesity are a growing problem in preschool children with 6.0% of the world’s children affected [8], low-resource regions such as South-eastern Asia, are reported to have the highest prevalence of overweight affecting 10.7% of under-five-year-olds [8]. The prevalence of overweight and obesity is even greater among school children in South-eastern Asia with more than 30% of children affected in some settings [9,10,11]. 

Childhood undernutrition also remains a key challenge for Vietnam. Although the prevalence of underweight in children under 5 years of age has decreased considerably from 39.0% in 1999 to 14.1% in 2015, the prevalence of stunting still remains high (24.6% in 2015) [7] and is of public health concern [12]. The increased prevalence of overweight and obesity among Vietnamese children is also concerning. While few children were obese in the late 20th century, by 2005 approximately 36.8% of preschool children have been affected [13]. However, anthropometric data in primary school children are limited. The most recent study, which included 2,872 children aged 5–11 years from 6 provinces in Vietnam, found that the prevalence of underweight and stunting was still high in both urban and rural areas (underweight: up to 25%; stunting: up to 17.7%), and that overweight/obesity affected up to 33.7% of children [14]. Prevalence data for undernutrition and overnutrition from school children residing in other provinces are still lacking.

Undernutrition is a public health concern as it is the main cause of child mortality [15,16] due to its role in reducing immune competence and thus increasing susceptibility to infections such as pneumonia, diarrhoea, or measles [16]. In contrast, childhood obesity has been identified as a risk factor for obesity in adulthood which in turn contributes to the development of cardiovascular disease, type 2 diabetes and its associated retinal and renal complications, asthma, infertility, psychiatric disorders, cancer, and other obesity-related disorders [17]. Clearly, this dual burden of diseases needs to be addressed and requires strong effort from the healthcare system [18]. However, to enable Vietnam’s limited resources to be allocated to the most vulnerable populations, research needs to identify children at higher risk of undernutrition and overnutrition. Therefore, the aims of this study were 1) to estimate the prevalence of undernutrition (i.e., underweight, stunting, wasting, and aggregate anthropometric failure) and overnutrition (i.e., overweight/obesity and abdominal overweight/obesity) among 6–9-year-old primary school children in rural areas in Hai Phong City, Vietnam, and 2) to identify sociodemographic factors associated with undernutrition and overnutrition in this population.

## 2. Materials and Methods

### 2.1. Study Design

This study used screening data from an intervention trial that investigated the effects of micronutrient supplementation for improving micronutrient status, growth, and health outcomes in underweight and overweight/obese primary school children in Vietnam (registered at www.actr.org.au as ACTRN12616001245482). A school-based cross-sectional survey of 6–9-year-old children (*n* = 2334) was conducted in eight primary schools in rural areas in Hai Phong City, Vietnam, in October 2016. Anthropometric measurements and self-reported sociodemographic data were collected. Ethical approval for the study was obtained from the Ethics Committee of the National Institute of Nutrition in Vietnam (610/VDD-QLKH; dated 30/9/2016) and the Deakin University Human Research Ethics Committee in Australia (2016-181). Written informed consent was obtained from the child’s primary caregiver and verbal consent was obtained from each participating child.

### 2.2. Schools Selection and Recruitment

Hai Phong City is administratively divided into eight rural districts and seven urban districts; this research was conducted in rural districts only. Schools located in rural areas of Hai Phong City were selected using a multistage sampling approach. In the first stage, two districts (Thuy Nguyen and An Lao) were randomly selected from the eight rural districts in Hai Phong City. In the second stage, schools were selected from the list of all primary schools in each district (*n* = 38 in Thuy Nguyen and *n* = 19 in An Lao). Schools with <300 students were excluded. Selected schools were invited to take part through written invitations followed by telephone calls, emails, and/or visits to explain the purpose of this study, confirm participation, and obtain written informed consent from school principals. When a school declined to take part in this study, the next school on the list was invited until four were enrolled in each district.

### 2.3. Participants and Recruitment 

Once a school agreed to participate, all children attending grades 1 to 3 (i.e., children aged 6–9 years) were invited to take part. Child’s date of birth was obtained from the school registration form in order to confirm the child’s age.

Children were excluded if they were older than 9 years, had anthropometric abnormalities (e.g., severe scoliosis which would not allow for correct determination of height), or had an intellectual impairment that would prevent them from understanding the aims of the study.

### 2.4. Anthropometric Measures

Children were weighed in light clothing and without shoes with calibrated electronic body scales (TANITA BC-543, TANITA Corporation, Tokyo, Japan). Each child was weighed twice to the nearest 0.1 kg; if the two measurements differed by more than 0.1 kg, a third measurement was taken. 

Height was measured twice to the nearest 0.1 cm with a SECA stadiometer (SECA 222, SECA GmbH & Co. KG, Hamburg, Germany). Children were measured without any hair ornaments, braids or hats, and without shoes or socks. If the duplicate measurements differed by more than 0.1 cm, a third measurement was taken.

Waist circumference (WC) was measured twice horizontally to the nearest 0.1 cm with a nonstretchable tape (Lufkin W606PM, Apex Tool Group, MD, USA) at a point halfway between the lowest rib and the top of a child’s hipbone [19]. If the two measurements differed by more than 0.1 cm, a third measurement was taken. 

The final weight, height, and WC were calculated as the mean of the two or three measurements obtained for each child, as appropriate. All anthropometric measurements were taken by trained staff from the National Institute of Nutrition in Vietnam according to standardised procedures [19].

Body Mass Index (BMI) was calculated. Weight-for-age, height-for-age, and BMI-for-age *z*-scores were calculated with the WHO Anthro Plus software version 2.0 [20]. Underweight, stunting, wasting, and overweight/obesity were defined according to the WHO classification [21], i.e., underweight: weight-for-age *z*-score < −2; stunting: height-for-age *z*-score < −2; wasting: BMI-for-age *z*-score < −2; and overweight/obesity: BMI-for-age *z*-score > 1; these categories were not exclusive and overlapped in some cases (e.g., a child who was classified as underweight might also be classified as stunted). An aggregate indicator of malnutrition, the Composite Index of Anthropometric Failure (CIAF), was determined by identifying all children who were in anthropometric failure (i.e., all children who were (1) wasted only; (2) wasted and underweight; (3) wasted, stunted, and underweight; (4) stunted and underweight; (5) stunted only; or (6) underweight only; wasting, stunting, and underweight were defined according to the WHO classification as described above) while excluding those children who were not in anthropometric failure (i.e., children whose weight-for-age, height-for-age, and BMI-for-age *z*-scores were ≥−2) [22]. The CIAF identifies children who belong exclusively to one of these categories; therefore, it provides a single measure of the severity of undernutrition in the population [22]. A waist-to-height ratio (WHtR) was calculated as WC in cm divided by height in cm. There are no specific WHtR cutoffs for the identification of overweight or obesity in Vietnamese children; therefore, we defined abdominal overweight/obesity as WHtR ≥ 0.46 for boys and WHtR ≥ 0.45 for girls according to the cutoffs developed by Nambiar et al. [23]. These cutoffs have been shown to correspond to the ≥ 85th percentile for percentage body fat (i.e., ≥ overweight) in a nationally representative sample of 2773 8–16-year-old Australian school children, which also included Asian children [23].

### 2.5. Socioeconomic Data

Mothers’ current employment status, mothers’ education level, and monthly household income data were collected from the mothers via a pretested self-administered questionnaire.

### 2.6. Data Analysis

Data were collected from 2334 children. Sex data were available for all children; weight data for 2333 children; age data for 2331 children; height, weight-for-age *z*-score, and CIAF data for 2330 children; WC data for 2329 children; height-for-age and BMI-for-age *z*-score data for 2327 children; and WHtR data for 2326 children. Seventy-one percent of mothers provided information on their current employment status (*n* = 1647), and their education level (*n* = 1652), and 68% of mothers (*n* = 1587) provided information on the monthly household income.

The prevalence of underweight, stunting, wasting, anthropometric failure, overweight/obesity, and abdominal overweight/obesity was estimated for the whole sample, and for the levels of the following factors: sex, age, mother’s current employment status, mother’s education level, and monthly household income.

Children’s age was categorized into tertiles (69 to <82 months, 82 to <93 months, and 93 to ≤108 months). Mothers’ education level was classified into three categories: beyond high school, high school, and below high school. High school education comprises 3 years of formal schooling, from the 10th to 12th grade, and is normally the minimum qualification required to obtain employment in Vietnam. The average income in rural areas in Vietnam in 2014 was 4.1 million VND/two adults/month (i.e., 176 USD) [24]. This monthly household income was used as a cutoff to differentiate between below-average and above-average income. Monthly household income was classified into four categories: <4.1 million VND (i.e., <176 USD), 4.1 to <8 million VND (i.e., 176 to <344 USD), 8 to <11 million VND (i.e., 344 to <472 USD), and ≥ 11 million VND (i.e., ≥472 USD).

Generalised linear mixed models, with school as a random effect, were used to estimate the association between binary outcomes (underweight, stunting, wasting, anthropometric failure, overweight/obesity, and abdominal overweight/obesity) with demographic variables. We report univariate associations, i.e., only one demographic factor considered at a time, and adjusted associations estimated under models including all demographic factors (i.e., age, sex, mother’s current employment status, mother’s education level, and monthly household income) [25]. Continuous outcomes (BMI-for-age *z*-scores and WHtR) were analysed following the same approach but using linear mixed models. For each outcome, we also report a measure of similarity of outcomes among children attending the same school, the intracluster correlation coefficient, estimated under a model with school as a random effect and no covariates. All estimates are reported along with 95% confidence intervals (CI). Analyses were performed with Stata (version 14.0; StataCorp LP, TX, USA).

## 3. Results

### 3.1. Participants

A total of 3960 children were invited to take part in this study. Of these, 2334 children were eligible and agreed to participate. A similar proportion of girls and boys took part in this study (Table 1). 

The majority of children’s mothers currently engaged in farm work and nearly two thirds were educated to a high school level or beyond (Table 1). Among the mothers who were self-employed, 84.7% had a low level of education (i.e., below high school) (data not shown in tables). More than half of the participating children came from households with a mean monthly income of ≥ 8 million VND, whereas nearly one in six children came from families with a mean monthly income below the average two-adult income in rural areas in Vietnam. Mothers from families with a below-average monthly income were more likely to have a low education level (67.9% had an education level below high school) (data not shown in tables).

### 3.2. Prevalence of Undernutrition and Overnutrition, and Associations between Anthropometric Indicators and Sociodemographic Factors

The prevalence of underweight was 8.0% (i.e., all these children were underweight, in addition, some children were also stunted, stunted and wasted, or wasted) with no significant difference between boys and girls, or across age or household income categories (Table 2). Mother’s current employment status and education level were univariately associated with underweight. In the multivariate analysis including all sociodemographic factors, mother’s education level was the only factor significantly associated with underweight. Children of mothers with low education (below high school) were more likely to be underweight compared with children whose mothers were educated beyond high school (Odds Ratio (OR) 3.60; 95% CI: 1.95, 6.64).

The prevalence of stunting was 5.1%. In univariate analyses, stunting was only associated with mother’s education level (Table 3). After adjustment for all sociodemographic factors, there was no factor significantly associated with stunting.

The prevalence of wasting was 5.3% with no significant differences between sexes, or across the child age or mother’s current employment status (Table 4). In univariate analyses, mother’s education level and monthly household income were significantly associated with wasting; however, these associations were no longer significant after adjustment for other sociodemographic factors.

The aggregate prevalence of undernutrition as measured by the CIAF indicated that 11.9% of all children were in anthropometric failure (Table 5). Anthropometric failure was significantly associated in univariate analyses with child age, mother’s current employment status, mother’s education level, and monthly household income. However, in the multivariate analysis, only mother’s education level was associated with anthropometric failure. Children whose mothers’ education level was below high school were more likely to be in anthropometric failure compared with children whose mothers were educated beyond high school (OR 2.00, 95% CI: 1.26, 3.19).

The prevalence of overweight/obesity was 22.1%, and it was higher in boys than girls (27.2% vs. 17.1%; *p* < 0.001; Table 6). Overweight/obesity was also significantly associated in the univariate analyses with mother’s current employment status, mother’s education level, and monthly household income. However, in the multivariate analysis only child sex and mother’s education level remained significantly associated with overweight/obesity. In comparison with children whose mothers had an education level above high school, children whose mothers had high school or below high school education were significantly less likely to be overweight/obese (OR 0.59, 95% CI: 0.44, 0.81, and OR 0.37, 95% CI: 0.26, 0.53, respectively; Table 6). Similar results were obtained when BMI-for-age *z*-scores were analysed (Table 7).

The prevalence of abdominal overweight/obesity was 31.0% (Table 8). In univariate analyses, abdominal overweight/obesity was significantly associated with sex, mother’s current employment status, mother’s education level, and monthly household income. In the multivariate model, only sex and mother’s education level were significantly associated with abdominal overweight/obesity, with a higher risk of abdominal overweight/obesity in boys compared with girls (OR 1.55, 95% CI: 1.24, 1.93), and a lower risk in children whose mothers’ education level was at or below high school compared with children whose mothers were educated beyond high school (OR 0.65, 95% CI: 0.49, 0.86 and OR 0.47, 95% CI: 0.35, 0.65, respectively). Additionally, WHtR was higher in boys than girls, lower in the oldest children compared with the youngest children, and lower in children of mothers with education at or below high school compared with children of mothers with education above high school (Table 9).

Finally, we present in Table 10 the estimated intracluster correlation coefficient for each of the outcomes assessed in this study. These estimates may inform the design and sample size calculations in future studies targeting the same outcomes in school children from rural areas in Vietnam.

## 4. Discussion

### 4.1. Prevalence of Undernutrition and Overnutrition

This study provides evidence for the double burden of disease in primary school children in rural areas of Hai Phong City, Vietnam with nearly 12% of children affected by anthropometric failure, 22% by overweight/obesity, and 31% by abdominal overweight/obesity.

We found that 8% of primary school children in rural areas in Hai Phong City in Vietnam were underweight. This confirms the results of our earlier cross-sectional study in 1004 primary school children in rural and urban areas of Hai Phong City in Vietnam which found 8.2% of children to be underweight [26].

Vietnam has been identified as one of 36 nations with the highest prevalence of stunting in the world [27]. In the present study, we found that 5.1% of children were at risk of stunting, which is similar to the prevalence of 7.6% found in our earlier cross-sectional study [26], or 2.4% reported recently in 3,000 primary school children in Hanoi City [28]. Compared with previous studies in other cities in Vietnam, wasting appeared less prevalent among primary school children in rural areas of Hai Phong City. Approximately 14% of primary school children in 6 provinces in Vietnam were affected by wasting in 2011 [14] compared with 5.3% children in our study. However, this proportion of children at risk of wasting has apparently not changed in the last four years as we also found that 5.3% of school children were wasted in our earlier cross-sectional study conducted in the same city in 2012 [26]. Hai Phong City is a mega city in Vietnam (population of approximately 2 million people in 2016) with better socioeconomic conditions compared to other regions of Vietnam [29], which could be at least partially responsible for the lower risk of wasting. 

A study conducted in 2011 in 2,872 children in 6 provinces in Vietnam found that 6.5% of school children in rural areas were affected by overweight and/or obesity [14]. In 2012, we found that 12% of school children in rural areas of Hai Phong City were affected by overweight or obesity [26]. In the current study, however, this prevalence nearly doubled as 22% of children were affected. This sharp increase in overweight and obesity in Hai Phong City over the last four years indicates that children in rural areas of Vietnam are at similar risk of excessive body weight as those residing in urban areas where a third of children are affected [13,14,30]. Improved socioeconomic status, poor diet, and inadequate physical inactivity may be partially responsible for this high increase in the prevalence of overweight and obesity in the rural areas of Hai Phong City [30,31]. Additionally, Vietnamese households have been shown to be susceptible to the marketing of energy-dense nutrient-poor foods [32]. Advertising of food products has been identified to affect food preferences, purchases, consumption [33], and food choices of children [34]. Marketing of energy-dense nutrient-poor foods, therefore, may contribute to childhood obesity [35].

### 4.2. Associations between Sociodemographic Factors and Anthropometric Indicators

This study revealed a significant and consistent relationship between maternal education and both undernutrition and overnutrition, and an association between sex and overnutrition. 

Indeed, we found that low maternal education was a risk factor for underweight and anthropometric failure. This is consistent with findings from studies in Vietnam [14,36] and other low- and middle-income countries [37,38]. However, no significant association between undernutrition and maternal employment status or monthly household income was detected after adjusting for other sociodemographic variables including education. This is in contrast with other studies, which reported a significant negative correlation between income and malnutrition [37,38,39]. It is possible that we did not detect an association between household income and undernutrition in our study because the majority of families (i.e., 83.9%) reported monthly income at or above the average two-adult income in rural areas of Vietnam, and only four families reported income at or below the poverty line of 0.4 million VND per month as defined by the Vietnamese Ministry of Labour, Invalids, and Social Affairs.

While undernutrition has been shown to be positively associated in rural areas with male sex [14] and age [39], we did not detect any association. Only in our univariate model age was associated with anthropometric failure, with older children at greater risk. Suboptimal habitual food consumption patterns and practices over the long term may be responsible for this trend [40]; however, our study did not explore this relationship. Similar to our research, a large study in Vietnam conducted in 2872 children found no relationship between undernutrition and child sex or age except for stunting, which was significantly higher in rural boys compared with girls (20.6% vs. 14.7%) [14].

Similar to undernutrition, we also found an association between overnutrition (i.e., overweight/obesity and abdominal overweight/obesity) and maternal education; however, this association was positive. Children of mothers with an education level above high school were at highest risk of overweight/obesity. This is consistent with findings from our earlier cross-sectional study in 276 primary school children in rural and urban areas of Hai Phong City [25] but in contrast with research conducted in other provinces/cities in Vietnam in which the education level of parents was protective against childhood overweight and obesity [41,42]. We have speculated that in Hai Phong City, women who achieved a higher education level could find better jobs resulting in less time spent preparing and providing healthier meals for children (for example, greater reliance on fast foods or processed foods) leading to a higher risk of childhood overweight/obesity [25]. Our current study also supports the suggestion of Lindsay et al. [43] that childhood obesity prevention programmes should focus on parental roles.

In addition, our research showed that the prevalence of overnutrition was significantly different between sexes with boys at a higher risk of overweight/obesity and abdominal overweight/obesity compared with girls. This is consistent with previous findings in Vietnam [14,25]. In Vietnam, communities typically prioritise the needs of boys compared with girls [44], which could result in decreased expectations of boys to contribute to household chores, and instead increasing their time spent in physical inactivity, and hence risk of overweight/obesity. Furthermore, although we did not collect dietary data in the current study, our earlier research showed that boys in Hai Phong City consumed a greater variety of foods than girls [25], which has been previously shown to be a risk factor for increased energy intake and adiposity [45]. Although this study focused only on anthropometric data in association with selected socioeconomic factors, it is clear that programmes aiming to reduce overweight/obesity among school children in Vietnam should take into account the higher risk of overweight/obesity in boys.

The study’s strengths include a random selection of the study districts and a large sample size. Additionally, all measurements were performed by well-trained staff from the National Institute of Nutrition in Vietnam to ensure high quality of data. However, as this was a cross-sectional study, it is difficult to detect temporal associations between undernutrition or overnutrition and the selected sociodemographic factors. Although more than 2000 children participated in this survey and the study sample was likely to be representative of all children attending primary schools in rural areas of Hai Phong City [26], it is unlikely that these children were representative of all primary school children in Vietnam [36,46]. In addition, as there are no specific WHtR cutoffs for the identification of abdominal overweight or obesity in Vietnamese children, we used the cutoffs by Nambiar et al. [23], which were developed for Australian children. However, there is still limited evidence on the most appropriate WHtR cutoffs for identifying abdominal overweight/obesity in Asian children, and those currently proposed are based on BMI rather than body fat [47,48].

## 5. Conclusions

In summary, undernutrition continues to be a public health problem in primary school children in rural areas in Hai Phong City affecting more than one in ten children. Overweight/obesity appears to have increased in the last four years with up to a third of children currently at risk. Low maternal education level was found to be a risk factor for underweight or anthropometric failure, whereas being male and high maternal education level was found to be a risk factor for overweight/obesity and abdominal overweight/obesity. Future interventions for the prevention and control of childhood obesity should take into consideration the difference in adiposity between boys and girls, and the need to target children of well-educated mothers. Programmes aimed at preventing child undernutrition need to emphasize the needs of children of mothers with low education.

## Figures and Tables

**Table 1:** Characteristics of the study participants.

All Children		*N* (2334)	%
Sex	Girls	1179	50.5
	Boys	1155	49.5
Age (months)	69 to <82	773	33.2
	82 to <93	806	34.6
	93 to ≤108	752	32.3
Mother’s current employment status	Self-employed	204	12.4
	Employed full time	342	20.8
	Farmer	970	58.9
	Unemployed	131	8.0
Mother’s education level	Above high school	465	28.1
	High school	554	33.5
	Below high school	633	38.3
Monthly household income (million VND)	<4.1	255	16.1
	4.1 to <8	501	31.6
	8 to <11	624	39.3
	≥11	207	13.0
Subgroups of anthropometric failure ^b^	No anthropometric failure ^a^	2053	88.1
	Composite Index of Anthropometric Failure	277	11.9
	Wasting only	60	2.6
	Wasting + underweight	51	2.2
	Wasting + stunting + underweight	13	0.6
	Stunting + underweight	74	3.2
	Stunting only	31	1.3
	Underweight only	48	2.1

^a^ Weight-for-age, height–for-age, and BMI-for-age *z*-scores ≥ −2. ^b^ Subgroups were defined according to the WHO classification for wasting, underweight, and stunting [21].

**Table 2:** Prevalence of underweight (weight-for-age *z*-score < −2) and associated sociodemographic factors in 6–9-year-old children in rural areas of Vietnam.

		Raw Estimates		Univariate Analysis ^a^			Multivariate Analysis ^b^ (*N* = 1574)		
		Total (*N*)	Underweight *N* (%)	OR (95% CI)	*p*-Value	Overall *p*-Value ^c^	OR (95% CI)	*p*-Value	Overall *p*-Value ^c^
All children		2330	186 (8.0)						
Sex	Girls (reference)	1177	91 (7.7)	1			1		
	Boys	1153	95 (8.2)	1.07 (0.79,1.45)	0.648		0.97 (0.67, 1.42)	0.878	
Age (months)	69 to <82 (reference)	773	57 (7.4)	1		0.434	1		0.557
	82 to <93	806	62 (7.7)	1.09 (0.75, 1.59)	0.656		0.97 (0.60, 1.57)	0.897	
	93 to ≤108	752	67 (8.9)	1. 27 (0.88, 1.85)	0.207		1.22 (0.77, 1.93)	0.398	
Mother’s current employment status	Self-employed (reference)	204	28 (13.7)	1		0.002	1		0.229
	Employed full time	341	20 (5.9)	0.39 (0.21, 0.72)	0.002		0.52 (0.27, 1.00)	0.052	
	Farmer	969	65 (6.7)	0.45 (0.28, 0.72)	0.001		0.67 (0.38, 1.19)	0.182	
	Unemployed	131	15 (11.5)	0.81 (0.42, 1.59)	0.544		0.90 (0.43, 1.89)	0.785	
Mother’s education level	Above high school (reference)	465	16 (3.4)	1		0.008	1		<0.001
	High school	553	33 (6.0)	1.28 (0.66, 2.50)	0.469		1.69 (0.90, 3.18)	0.108	
	Below high school	632	80 (12.6)	2.32 (1.27, 4.24)	<0.001		3.60 (1.95, 6.64)	<0.001	
Monthly household income (million VND)	<4.1 (reference)	254	24 (9.1)	1		0.060	1		0.073
	4.1 to <8	501	41 (8.2)	0.86 (0.51, 1.46)	0.575		1.12 (0.64, 1.94)	0.708	
	8 to <11	623	51 (8.2)	0.86 (0.52, 1.42)	0.551		1.67 (0.93, 3.00)	0.087	
	≥11	207	6 (2.9)	0.29 (0.12, 0.72)	0.008		0.66 (0.25, 1.75)	0.402	

^a^ Generalised linear mixed model including the covariate as a fixed effect and school as a random effect. ^b^ Generalised linear mixed model including all five sociodemographic factors as fixed effects and school as a random effect. ^c^ Overall *p*-value for the association between the outcome and the sociodemographic factor.

**Table 3:** Prevalence of stunting (height-for-age *z*-score < −2) and associated sociodemographic factors in 6–9-year-old children in rural areas of Vietnam.

		Raw Estimates		Univariate Analysis ^a^			Multivariate Analysis ^b^ (*N* = 1570)		
		Total (*N*)	Stunting *N* (%)	OR (95% CI)	*p*-Value	Overall *p*-Value ^c^	OR (95% CI)	*p*-Value	Overall *p*-Value ^c^
All children		2327	118 (5.1)						
Sex	Girls (reference)	1175	54 (4.6)	1			1		
	Boys	1152	64 (5.6)	1.22 (0.84, 1.77)	0.295		1.30 (0.82, 2.05)	0.267	
Age (months)	69 to <82 (reference)	773	34 (4.4)	1		0.117	1		0.100
	82 to <93	806	36 (4.5)	1.03 (0.64, 1.68)	0.893		1.12 (0.61, 2.05)	0.713	
	93 to ≤108	752	48 (6.4)	1.52 (0.96, 2.41)	0.072		1.77 (1.00, 3.13)	0.052	
Mother’s current employment status	Self-employed (reference)	204	15 (7.4)	1		0.265	1		0.594
	Employed full time	340	14 (4.1)	0.53 (0.25, 1.13)	0.103		0.63 (0.27, 1.43)	0.267	
	Farmer	968	45 (4.6)	0.61 (0.33, 1.11)	0.109		0.77 (0.37, 1.59)	0.477	
	Unemployed	131	9 (6.9)	0.89 (0.37, 2.13)	0.791		1.07 (0.44, 2.66)	0.882	
Mother’s education level	Above high school (reference)	465	15 (3.2)	1		0.008	1		0.055
	High school	553	23 (4.2)	1.28 (0.66, 2.49)	0.471		1.18 (0.59, 2.37)	0.647	
	Below high school	630	45 (7.1)	2.31 (1.26, 4.22)	0.007		2.05 (1.03, 4.04)	0.040	
Monthly household income (million VND)	<4.1 (reference)	254	13 (5.1)	1		0.202	1		0.143
	4.1 to <8	500	27 (5.4)	1.03 (0.52, 2.05)	0.930		1.28 (0.63, 2.62)	0.498	
	8 to <11	622	36 (5.8)	1.10 (0.57, 2.13)	0.778		1.74 (0.82, 3.70)	0.146	
	≥11	207	4 (1.9)	0.35 (0.11, 1.07)	0.074		0.60 (0.18, 2.04)	0.417	

^a^ Generalised linear mixed model including the covariate as a fixed effect and school as a random effect. ^b^ Generalised linear mixed model including all five sociodemographic factors as fixed effects and school as a random effect. ^c^ Overall *p*-value for the association between the outcome and the sociodemographic factor.

**Table 4:** Prevalence of wasting (BMI-for-age *z*-score < −2) and associated sociodemographic factors in 6–9-year-old children in rural areas of Vietnam.

		Raw Estimates		Univariate Analysis ^a^			Multivariate Analysis ^b^ (*N* = 1570)		
		Total (*N*)	Wasting *N* (%)	OR (95% CI)	*p*-Value	overall *p*-Value ^c^	OR (95% CI)	*p*-Value	Overall *p*-Value ^c^
All children		2327	124 (5.3)						
Sex	Girls (reference)	1175	65 (5.5)	1			1		
	Boys	1152	59 (5.1)	0.92 (0.64, 1.32)	0.656		1.30 (0.82, 2.05)	0.267	
Age (months)	69 to <82 (reference)	773	37 (4.8)	1		0.209	1		0.500
	82 to <93	806	38 (4.7)	0.99 (0.62, 1.58)	0.966		0.76 (0.43, 1.35)	0.351	
	93 to ≤108	752	49 (6.5)	1.39 (0.89, 2.17)	0.143		1.05 (0.61, 1.81)	0.851	
Mother’s current employment status	Self-employed (reference)	204	17 (8.3)	1		0.234	1		0.592
	Employed full time	340	17 (5.0)	0.58 (0.29, 1.16)	0.126		0.94 (0.44, 2.04)	0.885	
	Farmer	968	46 (4.7)	0.55 (0.31, 0.98)	0.042		0.99 (0.50, 2.00)	0.998	
	Unemployed	131	7 (5.3)	0.62 (0.25, 1.54)	0.304		0.49 (0.15, 1.54)	0.222	
Mother’s education level	Above high school (reference)	465	14 (3.0)	1		0.008	1		0.250
	High school	553	27 (4.9)	1.65 (0.86, 3.19)	0.134		1.42 (0.71, 2.83)	0.317	
	Below high school	630	46 (7.3)	2.54 (1.38, 4.68)	0.003		1.80 (0.90, 3.63)	0.098	
Monthly household income (million VND)	<4.1 (reference)	254	19 (7.5)	1		0.031	1		0.227
	4.1 to <8	500	32 (6.4)	0.85 (0.47, 1.53)	0.582		0.94 (0.51, 1.75)	0.853	
	8 to <11	622	22 (3.5)	0.45 (0.24, 0.85)	0.014		0.54 (0.27, 1.12)	0.098	
	≥11	207	7 (3.3)	0.43 (0.18, 1.05)	0.064		0.56 (0.22, 1.48)	0.243	

^a^ Generalised linear mixed model including the covariate as a fixed effect and school as a random effect. ^b^ Generalised linear mixed model including all five sociodemographic factors as fixed effects and school as a random effect. ^c^ Overall *p*-value for the association between the outcome and the sociodemographic factor.

**Table 5:** Prevalence of anthropometric failure and associated sociodemographic factors in 6–9-year-old children in rural areas of Vietnam.

		Raw Estimates		Univariate Analysis ^a^			Multivariate Analysis ^b^ (*N* = 1572)		
		Total(*N*)	Failure ^c^ *N* (%)	OR (95%CI)	*p*-Value	Overall *p*-Value ^d^	OR (95%CI)	*p*-Value	Overall *p*-Value ^d^
All children		2330	277 (11.9)						
Sex	Girls (reference)	1177	134 (11.4)	1			1		
	Boys	1153	143 (12.4)	1.09 (0.85, 1.40)	0.499		1.08 (0.79, 1.48)	0.612	
Age (months)	69 to <82 (reference)	773	78 (10.1)	1		0.005	1		0.068
	82 to <93	806	87 (10.8)	1.12 (0.81, 1.55)	0.509		1.02 (0.68, 1.53)	0.919	
	93 to ≤108	752	112 (14.9)	1.61 (1.18, 2.21)	0.003		1.48 (1.01, 2.17)	0.044	
Mother’s current employment status	Self-employed (reference)	204	36 (17.7)	1		0.028	1		0.623
	Employed full time	341	34 (10.0)	0.52 (0.31, 0.86)	0.010		0.69 (0.40, 1.20)	0.174	
	Farmer	969	104 (10.7)	0.56 (0.37, 0.85)	0.006		0.82 (0.50, 1.34)	0.427	
	Unemployed	131	19 (14.5)	0.79 (0.43, 1.45)	0.449		0.82 (0.42, 1.59)	0.548	
Mother’s education level	Above high school (reference)	465	34 (7.3)	1		<0.001	1		0.004
	High school	553	55 (10.0)	1.40 (0.89, 2.18)	0.142		1.21 (0.76, 1.94)	0.423	
	Below high school	632	105 (16.6)	2.53 (1.68, 3.79)	<0.001		2.00 (1.26, 3.19)	0.004	
Monthly household income (million VND)	<4.1 (reference)	254	35 (13.8)	1		0.019	1		0.313
	4.1 to <8	501	70 (14.0)	1.02 (0.66, 1.58)	0.918		1.24 (0.78, 1.96)	0.366	
	8 to <11	623	66 (10.6)	0.74 (0.48, 1.15)	0.185		1.11 (0.67, 1.82)	0.687	
	≥11	207	13 (6.3)	0.42 (0.22, 0.82)	0.011		0.69 (0.34, 1.42)	0.315	

^a^ Generalised linear mixed model including the covariate as a fixed effect and school as a random effect. ^b^ Generalised linear mixed model including all five sociodemographic factors as fixed effects and school as a random effect. ^c^ Composite Index of Anthropometric Failure; weight-for-age, height-for-age, and/or BMI-for-age *z*-scores < −2. ^d^ Overall p-value for the association between the outcome and the sociodemographic factor.

**Table 6:** Prevalence of overweight/obesity (BMI-for-age *z*-score > 1) and associated sociodemographic factors in 6–9-year-old children in rural areas of Vietnam.

		Raw Estimates		Univariate Analysis ^a^			Multivariate Analysis ^b^ (*N* = 1570)		
		Total(*N*)	Overweight/Obesity *N* (%)	OR (95% CI)	*p*-Value	Overall *p*-Value ^c^	OR (95% CI)	*p*-Value	Overall *p*-Value ^c^
All children		2327	514 (22.1)						
Sex	Girls (reference)	1175	201 (17.1)	1			1		
	Boys	1152	313 (27.2)	1.83 (1.50, 2.25)	<0.001		2.04 (1.58, 2.61)	<0.001	
Age (months)	69 to <82 (reference)	773	150 (19.5)	1		0.146	1		0.071
	82 to <93	806	197 (24.5)	1.28 (1.00, 1.63)	0.050		1.41 (1.05, 1.90)	0.023	
	93 to ≤108	752	167 (22.2)	1.16 (0.90, 1.49)	0.253		1.16 (0.84, 1.60)	0.368	
Mother’s current employment status	Self-employed (reference)	204	29 (14.2)	1		0.022	1		0.647
	Employed full time	340	82 (24.1)	1.80 (1.12, 2.88)	0.015		1.19 (0.70, 2.03)	0.528	
	Farmer	968	221 (22.8)	1.66 (1.09, 2.55)	0.020		0.97 (0.59, 1.62)	0.920	
	Unemployed	131	20 (15.3)	1.02 (0.54, 1.88)	0.959		0.96 (0.49, 1.87)	0.894	
Mother’s education level	Above high school (reference)	465	147 (31.6)	1		<0.001	1		<0.001
	High school	553	117 (21.2)	0.58 (0.44, 0.77)	<0.001		0.59 (0.44, 0.81)	0.001	
	Below high school	630	89 (14.1)	0.36 (0.26, 0.48)	<0.001		0.37 (0.26, 0.53)	<0.001	
Monthly household income (million VND)	<4.1 (reference)	254	37 (14.6)	1		0.003	1		0.561
	4.1 to <8	500	92 (18.4)	1.31 (0.86, 1.99)	0.208		1.12 (0.72, 1.73)	0.620	
	8 to <11	622	154 (24.8)	1.86 (1.25, 2.78)	0.002		1.30 (0.84, 2.02)	0.239	
	≥11	207	56 (27.1)	2.02 (1.25, 3.26)	0.004		1.35 (0.80, 2.27)	0.256	

^a^ Generalised linear mixed model including the covariate as a fixed effect and school as a random effect. ^b^ Generalised linear mixed model including all five sociodemographic factors as fixed effects and school as a random effect. ^c^ Overall *p*-value for the association between the outcome and the sociodemographic factor.

**Table 7:** BMI-for-age *z*-scores and associated sociodemographic factors in 6–9-year-old children in rural areas of Vietnam.

		Raw Estimates			Univariate Analysis ^a^			Multivariate Analysis ^b^ (*N* = 1570)		
		Total (*N*)	Mean	SE	β (95% CI)	*p*-Value	Overall *p*-Value ^c^	β (95% CI)	*p*-Value	Overall *p*-Value ^c^
All children		2327	−0.103	0.029						
Sex	Girls (reference)	1175	−0.293	0.101						
	Boys	1152	0.089	0.101	0.382 (0.272, 0.493)	<0.001		0.423 (0.288, 0.557)	<0.001	
Age (months)	69 to <82 (reference)	773	−0.116	0.105			0.206			0.106
	82 to <93	806	−0.035	0.104	0.080 (−0.057, 0.218)	0.251		0.153 (−0.009, 0.316)	0.065	
	93 to ≤108	752	−0.157	0.104	−0.042 (−0.182, 0.099)	0.560		0.001 (−0.167, 0.170)	0.991	
Mother’s current employment status	Self-employed (reference)	204	−0.463	0.128			<0.001			0.325
	Employed full time	340	0.007	0.115	0.470 (0.229, 0.712)	<0.001		0.244 (−0.018, 0.505)	0.068	
	Farmer	968	−0.038	0.095	0.425 (0.215, 0.635)	<0.001		0.145 (−0.098, 0.388)	0.243	
	Unemployed	131	−0.277	0.148	0.186 (−0.119, 0.490)	0.232		0.160 (−0.158, 0.479)	0.323	
Mother’s education level	Above high school (reference)	465	0.251	0.081			<0.001			<0.001
	High school	553	−0.072	0.077	−0.322 (−0.494, −0.150)	<0.001		−0.281 (−0.460, −0.101)	0.002	
	Below high school	630	−0.405	0.074	−0.656 (−0.824, −0.487)	<0.001		−0.577 (−0.770, −0.383)	<0.001	
Monthly household income (million VND)	<4.1 (reference)	254	−0.329	0.114			<0.001			0.422
	4.1 to <8	500	−0.224	0.096	0.105 (−0.105, 0.316)	0.327		−0.036 (−0.249, 0.178)	0.744	
	8 to <11	622	0.016	0.092	0.345 (0.140, 0.550)	0.001		0.070 (−0.152, 0.292)	0.537	
	≥11	207	0.131	0.121	0.460 (0.202, 0.719)	<0.001		0.147 (−0.127, 0.422	0.293	

^a^ Linear mixed model including the covariate as a fixed effect and school as a random effect. ^b^ Linear mixed model including all five sociodemographic factors as fixed effects and school as a random effect. ^c^ Overall *p*-value for the association between the outcome and the sociodemographic factor.

**Table 8:** Prevalence of abdominal overweight/obesity (WHtR ≥ 0.46 for boys and ≥ 0.45 for girls) and associated sociodemographic factors in 6–9-year-old children in rural areas of Vietnam.

		Raw Estimates		Univariate Analysis ^a^			Multivariate Analysis ^b^ (*N* = 1567)		
		Total(*N*)	Abdominal Overweight/ Obesity *N* (%)	OR (95% CI)	*p*-Value	Overall *p*-Value ^c^	OR (95% CI)	*p*-Value	Overall *p*-Value ^c^
All children		2326	721 (31.0)						
Sex	Girls (reference)	1174	325 (27.7)	1			1		
	Boys	1152	396 (34.4)	1.37 (1.15, 1.64)	<0.001		1.55 (1.24, 1.93)	<0.001	
Age (months)	69 to <82 (reference)	773	260 (33.9)	1		0.070	1		0.180
	82 to <93	806	246 (30.6)	0.83 (0.67, 1.03)	0.092		0.94 (0.73, 1.22)	0.646	
	93 to ≤108	752	215 (28.6)	0.78 (0.62, 0.97)	0.028		0.78 (0.59, 1.02)	0.072	
Mother’s current employment status	Self-employed (reference)	204	44 (21.6)	1		0.021	1		0.876
	Employed full time	341	110 (32.3)	1.67 (1.11, 2.51)	0.014		1.19 (0.75, 1.87)	0.459	
	Farmer	966	316 (32.7)	1.70 (1.18, 2.45)	0.004		1.09 (0.71, 1.67)	0.700	
	Unemployed	131	34 (26.0)	1.25 (0.75, 2.11)	0.389		1.06 (0.61, 1.85)	0.829	
Mother’s education level	Above high school (reference)	463	193 (41.7)	1		<0.001	1		<0.001
	High school	553	167 (30.2)	0.61 (0.47, 0.78)	<0.001		0.65 (0.49, 0.86)	0.003	
	Below high school	631	146 (23.1)	0.42 (0.32, 0.55)	<0.001		0.47 (0.35, 0.65)	<0.001	
Monthly household income (million VND)	<4.1 (reference)	255	59 (23.1)	1		0.009	1		0.772
	4.1 to <8	499	144 (28.9)	1.34 (0.94, 1.90)	0.102		1.16 (0.80, 1.68)	0.426	
	8 to <11	621	211 (34.0)	1.68 (1.19, 2.36)	0.003		1.22 (0.84, 1.78)	0.293	
	≥11	207	74 (35.8)	1.80 (1.19, 2.72)	0.005		1.20 (0.76, 1.88)	0.420	

^a^ Generalised linear mixed model including the covariate as a fixed effect and school as a random effect. ^b^ Generalised linear mixed model including all five sociodemographic factors as fixed effects and school as a random effect. ^c^ Overall *p*-value for the association between the outcome and the sociodemographic factor.

**Table 9:** Waist-to-Height Ratio and associated socio-demographic factors in 6–9-year-old children in rural areas of Vietnam.

		Raw Estimates			Univariate Analysis ^a^			Multivariate Analysis ^b^ (*N* = 1567)		
		Total (*N*)	Mean	SE	β (95% CI)	*p*-Value	Overall *p*-Value ^c^	β (95% CI)	*p*-Value	Overall *p*-Value ^c^
All children		2326	0.444	0.001						
Sex	Girls (reference level)	1174	0.434	0.003						
	Boys	1152	0.452	0.003	0.018 (0.015, 0.022)	<0.001		0.020 (0.016, 0.025)	<0.001	
Age (months)	69 to <82 (reference)	773	0.448	0.003			0.002			0.006
	82 to <93	806	0.443	0.003	−0.004 (−0.009, −0.000)	0.048		−0.001 (−0.008, 0.002)	0.236	
	93 to ≤108	752	0.439	0.003	−0.008 (−0.013, −0.004)	<0.001		−0.009 (−0.014, −0.003)	0.001	
Mother’s current employment status	Self-employed (reference level)	204	0.436	0.004			0.037			0.876
	Employed full time	341	0.445	0.003	0.009 (0.001, 0.017)	0.020		0.003 (−0.006, 0.011)	0.545	
	Farmer	966	0.444	0.002	0.009 (0.002, 0.015)	0.012		0.000 (−0.007, 0.008)	0.910	
	Unemployed	131	0.438	0.004	0.003 (−0.007, 0.012)	0.607		0.002 (−0.009, 0.012)	0.764	
Mother’s education level	Above high school (reference level)	463	0.453	0.002			<0.001			<0.001
	High school	553	0.442	0.002	−0.010 (−0.016, −0.005)	<0.001		−0.009 (−0.015, −0.003)	0.002	
	Below high school	631	0.436	0.002	−0.017 (−0.022, −0.011)	<0.001		−0.014 (−0.021, −0.008)	<0.001	
Monthly household income (million VND)	<4.1 (reference level)	255	0.436	0.003			0.006			0.588
	4.1 to <8	499	0.440	0.003	0.004 (−0.003, 0.011)	0.283		0.000 (−0.007, 0.007)	0.981	
	8 to <11	621	0.447	0.002	0.010 (0.004, 0.017)	0.002		0.004 (−0.003, 0.011)	0.315	
	≥11	207	0.446	0.003	0.010 (0.001, 0.018)	0.021		0.002 (−0.007, 0.011)	0.693	

^a^ Linear mixed model including the covariate as a fixed effect and school as a random effect. ^b^ Linear mixed model including all five sociodemographic factors as fixed effects and school as a random effect. ^c^ Overall *p*-value for the association between the outcome and the sociodemographic factor.

**Table 10:** Estimates of the intracluster correlation coefficient (ICC) for each outcome measure ^a^.

	ICC (95% CI)
Underweight ^b^	0.013 (0.001, 0.116)
Stunting ^c^	0.008 (0.000, 0.349)
Wasting ^d^	0.001 (0.000, 0.999)
Overweight/obesity ^e^	0.038 (0.012, 0.113)
Abdominal overweight/obesity ^f^	0.020 (0.006, 0.068)
Composite Index of Anthropometry Failure ^g^	0.009 (0.001, 0.091)
BMI-for-age *z*-score	0.034 (0.012, 0.096)
Waist-to-Height Ratio	0.019 (0.006, 0.061)

^a^ ICC estimated under a mixed model with school as a random effect and no covariates. ^b^ Weight-for-age *z*-score < −2. ^c^ Height-for-age *z*-score < −2. ^d^ BMI-for-age *z*-score < −2. ^e^ BMI-for-age *z*-score > 1. ^f^ Waist-to-Height Ratio ≥ 0.46 for boys and ≥ 0.45 for girls. ^g^ Weight-for-age, height–for-age, and/or BMI-for-age *z*-scores < −2.
